# Mode of cell death induced by the HSP90 inhibitor 17-AAG (tanespimycin) is dependent on the expression of pro-apoptotic BAX

**DOI:** 10.18632/oncotarget.1419

**Published:** 2013-09-29

**Authors:** Marissa V Powers, Melanie Valenti, Susana Miranda, Alison Maloney, Suzanne A. Eccles, George Thomas, Paul A Clarke, Paul Workman

**Affiliations:** ^1^ Cancer Research UK Cancer Therapeutics Unit, The Institute of Cancer Research, London, UK; ^2^ Divisions of Cancer Biology and Clinical Studies, The Institute of Cancer Research, London, UK; ^3^ Present address: UCB Pharma, Slough, UK; ^4^ Present address: OHSU Knight Cancer Institute, Oregon Health and Science University, Portland, OR, USA

**Keywords:** 17-AAG, HSP90, BAX, Apoptosis, Colon cancer

## Abstract

Inhibitors of the molecular chaperone heat shock protein 90 (HSP90) are of considerable current interest as targeted cancer therapeutic agents because of the ability to destabilize multiple oncogenic client proteins. Despite their resulting pleiotropic effects on multiple oncogenic pathways and hallmark traits of cancer, resistance to HSP90 inhibitors is possible and their ability to induce apoptosis is less than might be expected. Using an isogenic model for BAX knockout in HCT116 human colon carcinoma cells, we demonstrate the induction of BAX-dependent apoptosis at pharmacologically relevant concentrations of the HSP90 inhibitor 17-AAG both *in vitro* and in tumor xenografts *in vivo*. Removal of BAX expression by homologous recombination reduces apoptosis *in vitro* and *in vivo* but allows a lower level of cell death via a predominantly necrotic mechanism. Despite reducing apoptosis, the loss of BAX does not alter the overall sensitivity to 17-AAG *in vitro* or *in vivo*. The results indicate that 17-AAG acts predominantly to cause a cytostatic antiproliferative effect rather than cell death and further suggest that BAX status may not alter the overall clinical response to HSP90 inhibitors. Other agents may be required in combination to enhance tumor-selective killing by these promising drugs. In addition, there are implications for the use of apoptotic endpoints in the assessment of the activity of molecularly targeted agents.

## INTRODUCTION

The molecular chaperone heat shock protein 90 (HSP90) is of particular interest as a therapeutic target in cancer owing to its role in maintaining the correct conformation and stability of a number of key oncogenic client proteins such as receptor and non-receptor tyrosine kinases (e.g. ERBB2, ALK, ABL) and serine/threonine kinases (e.g. CRAF, BRAF, AKT, CDK4), including especially those with oncogenic abnormalities and drug-resistant alleles [1, 2].

The natural product HSP90 inhibitors radicicol, geldanamycin and its derivative 17-allylamino-17-demethoxygeldanamycin (17-AAG) exert their effects by specifically interacting with the N-terminal ATP binding domain and inhibiting the intrinsic ATPase activity of HSP90 which is critical for its chaperone function [3-5]. Subsequently, HSP90 client proteins are degraded via the ubiquitin-proteasome pathway [6, 7].

We and others have previously demonstrated that 17-AAG induces depletion of key regulators of signal transduction in many human tumor models, including colon and breast cancer [8-10]. The subsequent inhibition of signal transduction pathways results in the induction of cytostasis and apoptosis, the extent of which is cancer cell line-dependent. We have shown previously in a small panel of four human colon adenocarcinomas that one tumor cell line, KM12, did not exhibit apoptosis in response to 17-AAG [8]. Unlike the other colon tumor cell lines in that study, including HCT116 cells, KM12 cells do not express the pro-apoptotic BCL2 family member BAX [8]. Based on that observation we hypothesized that BAX is required for the induction of apoptosis in response to 17-AAG. To test this we used an isogenic pair of HCT116 colon cancer cells, in one member of which BAX was knocked out using homologous recombination [11]. We found that following 17-AAG treatment BAX is required for the induction of cell death via the intrinsic apoptotic pathway. This was true for HCT116 cells in vitro and also the corresponding solid tumor xenografts growing in immune-compromised mice. In the absence of BAX in vitro total cell death was reduced but in the cell population that did undergo cell death, necrosis became the predominant mechanism. Interestingly, BAX knockout had no effect on the overall sensitivity of HCT116 cells when measured by SRB or MTT cell proliferation assays in vitro or on the response of HCT116 tumor xenografts in vivo. The results indicate that cytostatic effects on cell proliferation may represent the dominant therapeutic response in this cancer model. They also suggest that BAX status may not alter clinical response to HSP90 inhibitors and that other agents may be required in combination to enhance tumor-selective killing by these promising drugs. In addition, there are implications for the use of apoptotic endpoints in the assessment of the activity of molecularly targeted agents.

## RESULTS

### Confirmation of BAX status and effects of sulindac sulphide

**Figure 1:**
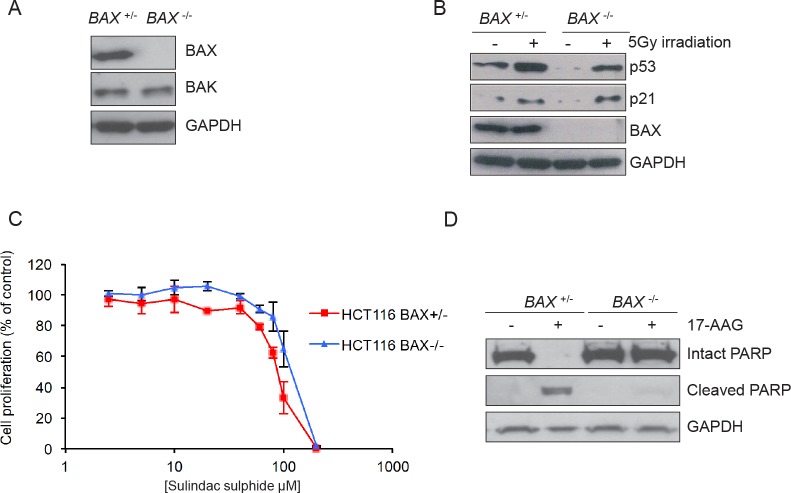
Validation of the isogenic model for BAX knockout in HCT116 human colon cancer cells (A) BAX is expressed in HCT116 *BAX* ^+/−^ but not in HCT116 *BAX* ^−/−^ cells. The cells were collected during logarithmic growth and analyzed for the presence of BAX and BAK by immunoblotting. (B) HCT116 *BAX* ^+/−^ and *BAX* ^−/−^ cells both showed induction of the p53 pathway in response to DNA damage. Cells were exposed to 5Gy irradiation and collected 4 hours after exposure. Expression of p53 and p21 was determined by immunoblotting. (C) BAX knockout does not affect sensitivity to sulindac sulfide when measured by 96 hours SRB cell proliferation assay following exposure to increasing concentrations of compound. Data are presented as mean ± SEM, N=3. (D) BAX knockout prevents apoptosis as determined by PARP cleavage in HCT116 *BAX* ^+/−^ and *BAX* ^−/−^ cells exposed to 2.5 × GI_50_ sulindac sulfide (HCT116 *BAX* ^+/−^ 233μM, HCT116 *BAX* ^−/−^ 273μM as determined by 96 hours SRB assay) or the equivalent concentration of drug vehicle. Cells were harvested after 48 hours and the expression of intact and cleaved PARP analyzed by immunoblotting. GAPDH was included as a loading control in panels A, B and D. Blots are representative of at least two independent experiments.

Previous reports have suggested that the loss of BAX is sufficient to confer resistance to apoptosis including that induced by several pharmacological agents [11]. To determine the role of BAX in 17-AAG induced apoptosis we used an isogenic pair of HCT116 human colon adenocarcinoma cell lines, as previously described [11]. HCT116 *BAX*^+/−^ cells are heterozygous for the *BAX* gene and express the BAX protein. In contrast, HCT116 *BAX* ^−/−^ cells have had the remaining *BAX* allele knocked out by homologous recombination resulting in complete loss of BAX protein expression, as confirmed here in Figure 1A. The isogenic cell line pair express similar levels of pro-apoptotic BAK and exhibit induction of p53 and p21 expression to a similar extent in response to 5Gy irradiation (Figure 1B).

HCT116 *BAX* ^−/−^ cells have been shown previously to be resistant to apoptosis induced by the non-steroidal anti-inflammatory drug sulindac sulfide, as determined by fluorescence microscopy of DAPI-stained cells and analysis of caspase-9 activation [11]. Interestingly, we observed no significant difference in sensitivity to sulindac sulfide between the two HCT116 isogenic cells types when assessed by 96 hours SRB assay to measure cell proliferation in the overall population (Figure 1C and Table 1; HCT116 *BAX* ^+/−^ 93.2μM ± 4.9 SEM, HCT116 *BAX* ^−/−^ 109.2μM ± 8.5 SEM *P* > 0.05).

**Table 1:** BAX status does not alter overall cellular sensitivity to sulindac sulphide or HSP90 inhibitors of different chemotypes. Exponentially growing HCT116 *BAX* ^+/−^ and *BAX* ^−/−^ cells were exposed to increasing concentrations of sulindac sulphide, 17-AAG, radicicol or CCT18159 for 96 hours and inhibition of cell proliferation was estimated using the SRB assay. An MTT assay was also used where stated. Concentration-response curves were plotted and the GI_50_ value determined as the compound concentration required to inhibit cell proliferation by 50% compared to drug vehicle treated controls. N = 3, data presented as the mean ± SEM. * N=2, data presented as the mean ± range

Compound	HCT116 BAX +/−	HCT116 BAX −/−
Sulindac	93.2μM ± 4.9	109.2μM ± 8.5
17-AAG	41.3nM ± 2.3	32.3nM ±1.3
17-AAG (MTT)	45.2nM ± 7.9	41.8nM ± 4.1
Radicicol	107nM ± 26.3	94.5nM ± 17.1
CCT18159*	5.3μM ± 0.5	5.5μM ± 0.1

The lack of significant difference in sensitivity of the overall tumor cell population to sulindac sulfide may be due to the SRB assay being a measure of total cellular protein (normally proportional to cell number remaining) present after 96-hour drug exposure, and hence does not distinguish between viable and nonviable cells or between cytostasis and cell death. HCT116 cells which become detached following 17-AAG treatment have previously been demonstrated to be apoptotic using morphology, sub-G1 distribution on flow cytometry and PARP cleavage analysis [8]. Therefore, to confirm that BAX expression influenced the apoptotic response to sulindac sulfide, detached cells were harvested after 48 hours exposure to 2.5×GI_50_ sulindac sulfide (as determined by 96 hours SRB assay) and the cleavage status of poly-ADP ribose polymerase (PARP) was analyzed by immunoblotting. PARP is a highly expressed nuclear protein that is a substrate for the apoptotic protease caspase-3 and PARP cleavage is a marker of apoptosis. Intact PARP was present in vehicle-treated control HCT116 *BAX* ^+/−^ cells whereas only the 89kDa caspase-3 cleaved form was detected in response to sulindac sulfide, consistent with apoptosis (Figure 1D). In contrast, only 116kDa intact PARP was detected in both vehicle and sulindac sulfide treated HCT116 *BAX* ^−/−^ cells (Figure 1D).

Our results are consistent with published data demonstrating that BAX expression is required for the induction of apoptosis in response to sulindac sulfide but also indicate that in HCT116 *BAX ^−/−^* cells a decrease in apoptotic response may not translate into increased sensitivity overall when measured by conventional cell proliferation assay [11].

### BAX knockout does not alter the overall cellular sensitivity to HSP90 inhibitors as measured by SRB and MTT assays

**Figure 2:**
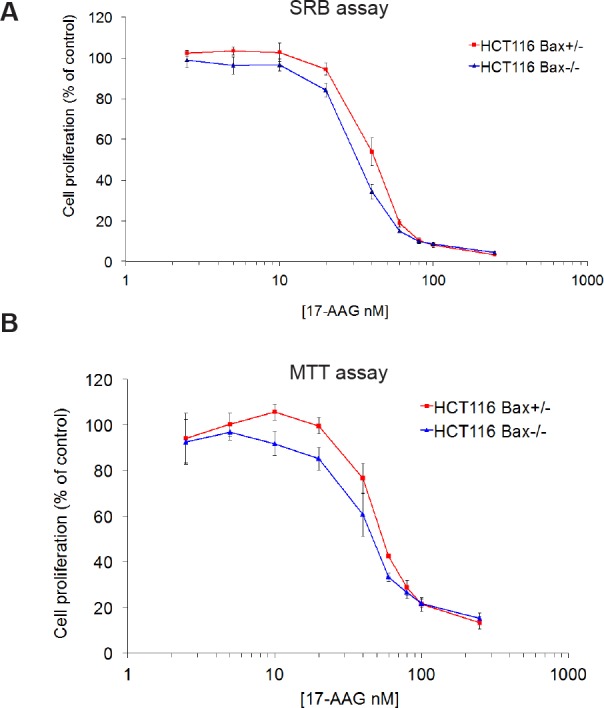
BAX knockout does not affect sensitivity to 17-AAG in HCT116 human colon cancer cells as measured by SRB or MTT assays Exponentially growing HCT116 *BAX* ^+/−^ and *BAX* ^−/−^ cells were exposed to increasing concentrations of 17-AAG for 96 hours and inhibition of cell proliferation was estimated using the (A) SRB assay or (B) MTT assay. Concentration-response curves were plotted and the GI_50_ value determined as the compound concentration required to inhibit cell proliferation by 50% compared to controls treated with drug vehicle. N=3, error bars indicate the mean ± SEM.

As seen with sulindac sulfide, 96 hour SRB cell proliferation assays with 17-AAG gave significantly similar GI_50_ values for both members of the HCT116 isogenic cancer cell line pair (Figure 2A and Table 1; HCT116 *BAX* ^+/−^ 41.3nM ± 2.3 SEM, HCT116 *BAX* ^−/−^ 32.3nM ±1.3 SEM, *P* > 0.05). Because of the possible discrepancy between measuring inhibition of cell proliferation by SRB and cell death, as seen above for sulindac sulfide, an MTT assay was also used. The MTT assay is based on the reduction of a tetrazolium salt by mitochondrial dehydrogenase [13]; therefore, it provides an indication of the number of viable cells remaining after 96 hours exposure to 17-AAG (Figure 2B). Consistent with the GI_50_ values determined for the isogenic pair using the SRB assay, no significant difference in the overall sensitivity to 17-AAG was observed by MTT assay between the two cell types (Figure 2B and Table 1; HCT116 *BAX* ^+/−^ 45.2nM ± 7.9 SEM, HCT116 *BAX* ^−/−^ 41.8nM ± 4.1 SEM *P* > 0.05). We also determined the sensitivity of the isogenic HCT116 cancer cell pair to the HSP90 inhibitors radicicol and CCT18159 [12], which are both chemically distinct from 17-AAG. Again, we observed no difference in the sensitivity of the isogenic cell line pair to these HSP90 inhibitors indicating that this lack of differential effect is not restricted to the benzoquinone ansamycin class of HSP90 inhibitors (Table 1). Thus BAX knockout does not affect the overall number of viable cells remaining 96 hours after HSP90 inhibition.

### 17-AAG treatment inhibits HSP90 function in both *BAX* ^+/−^ and *BAX* ^−/−^ HCT116 cells

It was important to establish that there was comparable inhibition of HSP90 in both members of the isogenic HCT116 cancer cell pair. Numerous studies have shown that inhibition of HSP90 activity by 17-AAG causes depletion of HSP90 client proteins CRAF and CDK4 [12, 16, 19, 20]. This is accompanied by the concurrent induction of HSP72, the inducible isoform of the HSP70 family, indicative of the HSF1-mediated heat shock response [12, 16, 19, 20]. Collectively, client protein depletion and HSP72 induction comprise a molecular biomarker signature of HSP90 inhibition that has been validated further in clinical trials [21-23].

**Figure 3:**
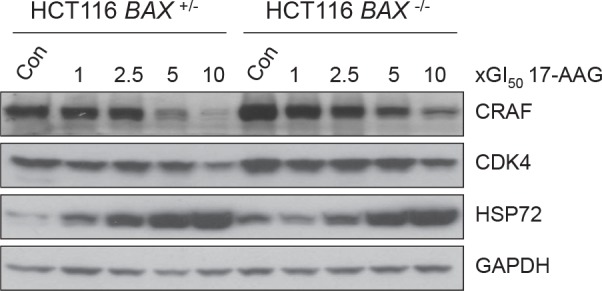
17-AAG shows similar inhibition of HSP90 regardless of BAX knockout in HCT116 human colon cancer cells, as determined by molecular biomarkers Exponentially growing HCT116 *BAX* ^+/−^ and *BAX* ^−/−^ cells were exposed to increasing multiples of the GI_50_ for 17-AAG (GI_50_; HCT116 *BAX* ^+/−^ 41.3nM ± 2.3 SEM, HCT116 *BAX* ^−/−^ 32.3nM ±1.3 SEM, as determined by 96 hours SRB assay). Cells were harvested 72 hours after the start of treatment and the expression levels of CRAF, CDK4 and HSP72 determined using immunoblotting. Controls (Con) were harvested at the time of treatment with 17-AAG. GAPDH was included as a loading control. Blots are representative of three independent experiments.

The expression levels of CRAF, CDK4 and HSP72 were analyzed by immunoblotting following 72 hours exposure to 17-AAG (Figure 3). In both *BAX* ^+/−^ and *BAX* ^−/−^ HCT116 cells CRAF was depleted and HSP72 induced in a concentration-dependent manner, the extent of which was generally similar in both cell types (Figure 3). CDK4 depletion was less than with CRAF, but was seen in both cell lines at 10 × GI_50_ 17-AAG (Figure 3)

### BAX knockout does not influence cell cycle distribution in response to 17-AAG, but does affect cell death

**Figure 4:**
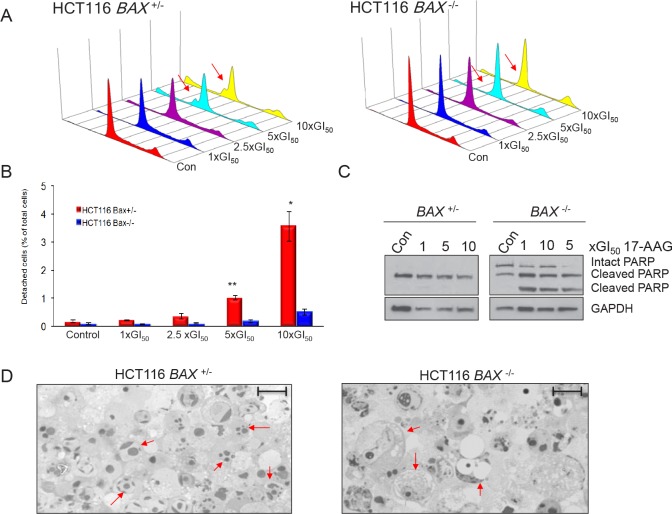
BAX knockout decreases apoptosis and increases necrosis in HCT116 human colon cancer cells treated with 17-AAG Exponentially growing HCT116 cells were exposed to increasing concentrations of 17-AAG at multiples of the GI_50_ for the individual cell types (HCT116 *BAX* ^+/−^ GI_50_ 41.3nM ± 2.3 SEM, HCT116 *BAX* ^−/−^ 32.3nM ±1.3 SEM, as determined by 96 hours SRB assay) or the equivalent volume of drug vehicle (DMSO). (A) BAX status alters the flow cytometry profile of HCT116 cells harvested 72 hours after the start of treatment. Cell cycle distribution was analyzed using propidium iodide staining. DNA Histograms are representative of two independent experiments. The sub-G1 peaks in HCT116 *BAX* ^+/−^ cells indicate apoptosis which was not seen in HCT116 *BAX* ^−/−^ cells. (B) BAX knockout reduces total cell death determined by quantifying the number of HCT116 *BAX* ^+/−^ and *BAX* ^−/−^ cells which became detached after 72 hours exposure to 17-AAG, quantified using a hemocytometer, * *P* < 0.05, ** *P* < 0.01. Data presented as mean ± SEM, N=3. (C) BAX status alters the mode of cell death as determined by analyzing the pattern of expression of PARP by immunoblotting in cells that had become detached following 17-AAG or DMSO exposure using an N-terminal specific antibody (C-2-10). GADPH was included as a loading control. Note that equal amounts of protein were loaded from the detached population in each case and hence the control populations also had detectable cleaved PARP (apoptotic or necrotic) that represented the background level of cell death for these cell types. (D) Morphological analysis confirms that BAX is required for apoptosis in response to 17-AAG treatment and necrosis occurs when BAX is absent. HCT116 *BAX* ^+/−^ and *BAX* ^−/−^ cells which were growing exponentially were treated with 5×GI_50_ 17-AAG for 72 hours. Detached cells were harvested and their morphology analyzed using toluidine blue staining. Arrows indicate apoptotic (HCT116 *BAX* ^+/−^) or necrotic (HCT116 *BAX* ^−/−^) characteristics. Images are representative of two independent experiments. Scale bar = 20μm

Although no significant difference was observed in the sensitivity of HCT116 *BAX* ^+/−^ and *BAX* ^−/−^ cells to 17-AAG using cell proliferation assays for the bulk population, it was demonstrated earlier using sulindac sulfide that these assays cannot be used to distinguish between a cytostatic or apoptotic response (Figure 1). It has previously been shown that parental HCT116 colon cancer cells respond to 17-AAG predominantly through a cytostatic mechanism [8, 20]. However, HCT116 cells do not exhibit accumulation at any particular phase of the cell cycle in response to 17-AAG [8]. Consistent with this, cell cycle analysis of HCT116 *BAX* ^+/−^ and *BAX* ^−/−^ cells showed no obvious accumulation within the G1-S or G2-M phases of the cell cycle in response to 72 hours exposure to increasing concentrations of 17-AAG (Figure 4A). However, sub-G1 populations were clearly and reproducibly detected in HCT116 *BAX* ^+/−^ cells at the higher concentrations of 17-AAG used (Figure 4A). In contrast, sub-G1 peaks were not detectable in HCT116 *BAX* ^−/−^ cells at any concentrations of 17-AAG investigated (Figure 4A). Sub-G1 peaks are characteristic of apoptotic cells, which are recognized as hypodiploid due to intra-nucleosomal degradation of DNA by endonucleases during the later stages of apoptosis. Therefore these data provided the first evidence to indicate that apoptotic cell death may occur in HCT116 *BAX* ^+/−^ but not isogenic BAX ^−/−^ cells in response to 17-AAG treatment.

As previously mentioned, HCT116 cells which become detached following treatment with 17-AAG are apoptotic according to their morphology, sub-G1 distribution on flow cytometry and PARP cleavage status [8]. Therefore, to further investigate the role of BAX in the cell death response to 17-AAG, the number of detached cells were counted 72 hours after the start of continuous exposure to 17-AAG (Figure 4B). We observed a concentration-dependent increase in the number of detached cells for both HCT116 *BAX* ^+/−^ and *BAX* ^−/−^ cancer cell lines (Figure 4B). However, although a concentration-dependent increase in the number of detached cells was seen in both members of the isogenic pair, the quantitative cell detachment response was much greater in HCT116 *BAX* ^+/−^ cells, which exhibited approximately 6.7 (± 1.5 SEM) and 7.6 (± 1.6 SEM) fold greater total cell death compared to HCT116 *BAX* knockout cells when treated with 5x and 10x GI_50_ 17-AAG respectively (*P* < 0.05; Figure 4B).

To investigate further whether the mechanism of cell death in the detached cells was apoptotic, the cleavage status of the apoptotic marker PARP was analyzed (Figure 4C). Consistent with our previous observations in parental HCT116 cells [8], HCT116 *BAX* ^+/−^ cells that had become detached following treatment with 17-AAG were confirmed as apoptotic by the detection of PARP in the 85kDa caspase-3 cleaved form (Figure 4C). Interestingly, although PARP cleavage was also found in isogenic HCT116 *BAX* ^−/−^ cells, the cleavage pattern was distinctly different from that associated with apoptosis (Figure 4C). In HCT116 *BAX* ^−/−^ cells PARP was present as three distinct bands corresponding to molecular weights of 116kDa, 85kDa and 62kDa (Figure 4C). This pattern of PARP cleavage, detected using an antibody specific for the N-terminal region of PARP, has previously been reported to be representative of necrotic cell death during which PARP is actively cleaved by lysosomal proteases (cathepsins) rather than caspases [26].

The apoptotic versus necrotic cleavage patterns of PARP observed in detached isogenic HCT116 *BAX* ^+/−^ and *BAX* ^−/−^ cells respectively (Figure 4C) were also observed in adherent cells following treatment with 17-AAG (supplemental Figure S1). However, the majority of PARP detected in the two adherent cell populations was in the intact form (supplemental Figure S1). This likely reflects a small population of apoptotic or necrotic cells, respectively, in *BAX* ^+/−^ and *BAX* ^−/−^ cells that are en route to detachment.

The predominant mechanisms of cell death induced by 17-AAG in HCT116 *BAX* ^+/−^ and *BAX* ^−/−^ cells was confirmed as apoptotic and necrotic respectively using toluidine blue staining (Figure 4D). Consistent with the PARP cleavage data described earlier (Figure 4C), characteristics indicative of apoptosis, including chromatin condensation [27, 28], were present in HCT116 *BAX* ^+/−^ but not isogenic *BAX* ^−/−^ cells. In contrast, HCT116 *BAX* ^−/−^ cells displayed cellular swelling and extensive cytoplasmic vacuolization in response to 17-AAG treatment (Figure 4D), both of which are indicative of necrosis [28].

### 17-AAG induces caspase-3 dependent apoptosis *in vivo*

**Figure 5:**
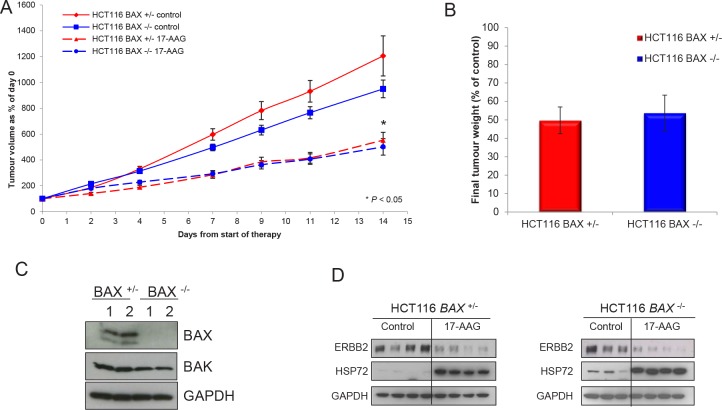
Overall response of HCT116 human colon cancer xenografts was independent of BAX status as measured by tumor volume and weight Tumor xenografts of the HCT116 *BAX* ^+/−^ and HCT116 *BAX* ^−/−^ human colon adenocarcinoma cell line were established s.c. bilaterally in the flanks of NCr athymic mice. (A) Animals received 80 mg/kg 17-AAG (or an equivalent volume of vehicle) i.p. on days 0-4, 7-11 and 14. Results are expressed as a percentage of tumor volumes at the start of therapy. Data presented as the mean ± SEM, N=16. *P < 0.05 relative to control. (B) Tumor weight determined after excision on day 5 in HCT116 *BAX* ^+/−^ and *BAX* ^−/−^ tumors following treatment with 17-AAG as above. Solid lines indicate vehicle treatment, broken lines indicate 17-AAG treatment. Data are expressed as a percentage of tumor weights in the vehicle treated control tumors ± SEM N=16. For analysis of BAX, BAK and biomarkers indicative of HSP90 inhibition, tumors were established as described above, treated with 80 mg/kg 17-AAG i.p. daily for four days and samples then taken 24 hours after the last dose. (C) Expression of BAX and BAK or (D) the biomarkers ERBB2 and HSP72 was determined by immunoblotting. GAPDH was included as a loading control. Numbers in panel C indicate two representative tumors in each case.

Next, we looked at the response of the isogenic cell pair when grown as solid tumor xenografts. Following five daily i.p doses of 80mg/kg 17-AAG to athymic mice bearing isogenic HCT116 *BAX* ^+/−^ or HCT116 *BAX* ^−/−^ human colon cancer xenografts, a clear effect on the growth of both tumor models was observed. A quantitatively similar reduction was observed in the mean tumor volume of both tumor xenografts (Figure 5A, *P* < 0.05). A very similar level of inhibition (HCT116 *BAX ^+/−^* 49.7% ± 7.2 SEM, HCT116 *BAX ^−/−^* 53.8% ± 9.7 SEM) was also demonstrated by the measurement of final tumor weights at the end of the experiment (Figure 5B).

Immunoblot analysis of tumor samples demonstrated that the isogenic status of the cell line pair was conserved in vivo with only the HCT116 *BAX* ^+/−^ line expressing BAX protein (Figure 5C). Consistent with the in vitro characterization shown earlier, both types of tumor expressed BAK (Figure 5C) and demonstrated comparable depletion of the HSP90 client protein ERBB2 and induction of HSP72 in response to 17-AAG treatment in vivo (Figure 5D) indicating comparable HSP90 target engagement in each member of the isogenic pair.

**Figure 6:**
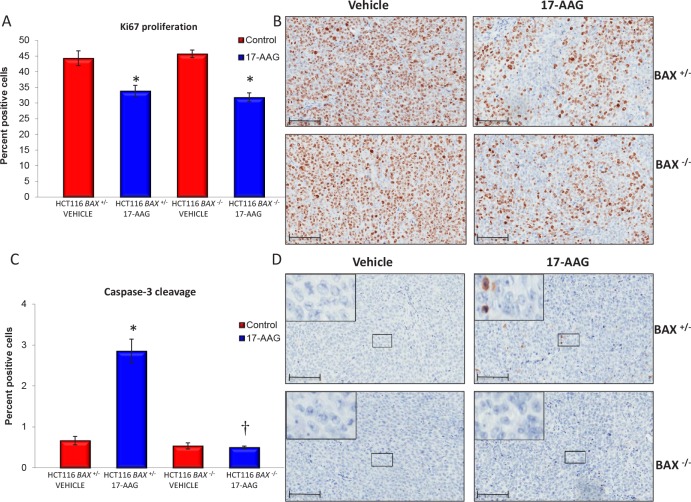
BAX knockout decreases caspase-3 dependent apoptosis in HCT116 human colon cancer xenografts Quantitative immunohistochemistry of HCT116 *BAX* ^+/−^ and isogenic *BAX* ^−/−^ tumor samples indicate increased apoptosis in *BAX* ^+/−^ tumors but similar reduction of proliferation in response to 17-AAG. Tumor xenografts of the HCT116 *BAX* ^+/−^ and HCT116 *BAX* ^−/−^ human colon adenocarcinoma cell line were established s.c. bilaterally in the flanks of NCr athymic mice. Animals received 80 mg/kg 17-AAG i.p. daily for four days and samples were taken 24 hours after the last dose. The proliferation marker Ki67 (A, B) or cleaved caspase-3 (C, D) were evaluated using quantitative immunohistochemistry following exposure to vehicle or 17-AAG. Data are presented as a percentage of total cells counted ± SEM, N=4, *P < 0.05, † *P* > 0.05 calculated relative to control. Representative images of Ki67 (B) or cleaved caspase-3 (D) staining are shown at x20 magnification; insert represents enlarged area shown on image. Brown staining indicates Ki67 or cleaved caspase-3 positive cells. Scale bars = 100μm

In line with the comparable effects on tumor growth and final tumor weights, 17-AAG treatment caused very similar decreases in the Ki67 staining for both HCT116 *BAX* ^+/−^ and HCT116 *BAX* ^−/−^ xenografts as determined on day five following daily i.p doses of 80mg/kg of the HSP90 inhibitor (Figure 6A and 6B). The decrease in Ki67 proliferation index is indicative of a marked antiproliferative effect of 17-AAG in both tumor models.

In contrast, and entirely consistent with our earlier in vitro findings, induction of apoptosis by 17-AAG was observed in HCT116 *BAX* ^+/−^ but not in HCT116 *BAX* ^−/−^ tumor xenografts as measured by the cleavage of caspase-3 on day five following daily i.p doses of 80mg/kg 17-AAG (Figure 6C and 6D). An approximate 4-fold induction of cells staining positive for caspase-3 cleavage was observed in HCT116 *BAX* ^+/−^ tumor xenografts, which was absent in isogenic HCT116 *BAX* ^−/−^ tumor xenografts (Figure 6C and 6D). Note, however, that the overall proportion of apoptotic cells remained low even in the treated tumors, increasing from 0.7% ± 0.1 SEM to 2.9% ± 0.3 SEM (Figure 6C and 6D).

These results show that, in line with the in vitro experiments, BAX knockout blocks the weak apoptotic effect of 17-AAG in HCT116 colon cancer xenografts. However, the overall inhibitory effects of 17-AAG on bulk tumor growth were comparable in the *BAX* ^+/−^ and *BAX* ^−/−^ HCT116 colon cancer models, indicating that apoptosis induction is not limiting in the in vivo solid tumor setting and that antiproliferative effects likely dominate the therapeutic response.

## DISCUSSION

Surprisingly given the depletion of multiple oncogenic proteins and blockade of many signaling pathways, it is well documented that HSP90 inhibition causes predominantly a cytostatic antiproliferative effect rather than cell death in various cancer cell lines [8, 20, 24, 25]. However, we previously demonstrated using a small panel of four human colon cancer cell lines that 17-AAG can also induce a degree of apoptotic cell death, the extent of which is tumor cell line-dependent [8]. In that previous study we observed that KM12 human colon carcinoma cells did not undergo significant apoptosis in response to 17-AAG [8]. Unlike the other colon cancer cell lines studied, KM12 cells do not express detectable levels of pro-apoptotic BAX [8]. Therefore, we hypothesized that the apoptotic response to HSP90 inhibition may be dependent on the expression of BAX. This was investigated in the present study using an isogenic pair of HCT116 human colon carcinoma cell lines that differed only in the expression of BAX.

Our analysis of the cell death response to 17-AAG revealed that HCT116 *BAX* ^+/−^ cells underwent approximately 6- to 7-fold greater apoptotic cell death than *BAX* ^−/−^ cells following exposure to pharmacologically relevant concentrations of 17-AAG. Flow cytometry analysis following 17-AAG treatment revealed the presence of sub-G1 peaks in HCT116 *BAX* ^+/−^ cells which were absent in isogenic *BAX* ^−/−^ cells. Sub-G1 peaks are indicative of DNA fragmentation which is associated with apoptotic cell death [29]. This apoptotic mode of cell death was confirmed in HCT116 *BAX* ^+/−^ cells by the detection of apoptotic morphology (i.e. condensed nuclei) and caspase-3 dependent cleaved PARP. Neither of these effects were detected in HCT116 *BAX* ^−/−^ cells following treatment with 17-AAG.

As observed consistently in the literature [8, 12, 20], we show here that inhibition of HSP90 by 17-AAG induces the expression of the anti-apoptotic molecular chaperone HSP72, as part of the HSF1-mediated heat shock response, together with degradation of several anti-apoptotic HSP90 client proteins, including CRAF and AKT [8, 12, 20]. Previous studies have demonstrated that the increased expression of HSP72 can provide a cytoprotective effect against the induction of apoptosis by inhibiting the oligomerisation of APAF-1 [30], activation of caspase-3 and −9 [30, 31] and antagonism of the activity of AIF [32]. HSP72 is known to interact with BAX to prevent its translocation from the cytoplasm to the mitochondria following heat shock and exposure to 17-AAG [33]. We and others have demonstrated that induction of HSP72 reduces the apoptotic response to 17-AAG [20, 33, 34] and silencing HSP72 expression sensitizes cells to the cell death effects of 17-AAG by promoting translocation of BAX to the mitochondria [33]. However, we show here that similar levels of HSP72 were induced in response to 17-AAG in isogenic HCT116 *BAX* ^+/−^ and *BAX* ^−/−^ cells. Therefore, it is unlikely that HSP72 was involved in the differential apoptotic response to 17-AAG observed between these two cell types.

The HSP90 client CRAF may also exert an anti-apoptotic role by its ability to modulate the activity of pro-apoptotic BAD [35]. Phosphorylation of BAD by CRAF has been reported to decrease the pro-apoptotic activity of this protein [35]. Thus the reduction of CRAF by 17-AAG may contribute to induction of apoptosis via reduced BCL2 activity. However, we show here that CRAF expression is reduced to a similar extent between HCT116 *BAX* ^+/−^ and *BAX* ^−/−^ cells in response to 17-AAG, indicating that the difference in BAX-mediated apoptosis is not due to differential CRAF degradation. Moreover, the similar depletion of CRAF and induction of HSP72 following 17-AAG treatment provide evidence of comparable HSP90 target engagement in the isogenic HCT116 cell line pair.

Interestingly, although we showed that apoptotic cell death in response to 17-AAG was blocked in the isogenic *BAX* knockout cells and the overall level of cell death was considerably reduced, we report here, to our knowledge for the first time, that the lower level of cell death that is observed in the *BAX* ^−/−^ cells occurs via a necrotic mechanism. This interesting switch in cell death mechanism was demonstrated by the presence of cellular swelling, extensive cytoplasmic vacuolization and detection of the necrotic signature of PARP cleavage in response to 17-AAG treatment. The necrotic signature includes a 62kDa fragment which has been shown to be a consequence of PARP cleavage by lysosomal proteases such as cathepsins, especially B and G [26]. These enzymes are released from the lysosome during necrosis allowing access to cytoplasmic or nuclear substrates [26]. Cleavage of PARP is considered to be a relatively early event associated with necrotic cell death and has been shown to occur before the widespread, non-specific DNA degradation that is associated with the final stages of necrosis [36].

Despite the block in apoptosis and greatly reduced total cell death in the absence of BAX, we observed that the overall *in vitro* sensitivity determined by SRB or MTT assays was no different between *BAX* ^+/−^ and *BAX* ^−/−^ HCT116 human colon cancer cells. The same lack of differential overall effect was seen with the chemically distinct HSP90 inhibitors radicicol and CCT18159 [12].

Similar to the above results measuring overall cell proliferation by SRB or MTT assays in vitro, we saw no difference in responsiveness to 17-AAG between *BAX* ^+/−^ and *BAX* ^−/−^ HCT116 cells when grown in vivo as solid tumor xenografts in immune-compromised mice, as measured by tumor volume or final tumor weight. This was despite a 4-fold induction of cleaved caspase-3 positive cells in *BAX* ^+/−^ HCT116 tumors, comparable to the increase seen in vitro, and a lack of apoptosis in *BAX* ^−/−^ HCT116 tumors, as also seen in cell culture. This suggests that the in vivo response of the bulk tumor is likely dictated by the drug-induced decrease in Ki67-positive proliferative cells which was similar in BAX ^+/−^ and BAX ^−/−^ HCT116 tumors. As seen before with the parental HCT116 cells [8] 17-AAG did not cause accumulation in any particular phase of the cell cycle in *BAX* ^+/−^ and *BAX* ^−/−^ HCT116 cells. This suggests that the antiproliferative effect is mediated by a general slowing of progression through the cell cycle in HCT116 cells.

Despite the clear differences in apoptosis induced by 17-AAG between HCT116 *BAX* ^+/−^ and isogenic *BAX* ^−/−^ cells, the proportion of apoptotic cells in HCT116 *BAX* ^+/−^ cells after 17-AAG treatment remained relatively small, accounting for only 3.6% of the total population in vitro and 2.9% in vivo. We conclude that the modest induction of apoptotic cell death observed in this study and elsewhere [8] in response to 17-AAG isreliant on BAX expression. However, the overall tumor response to 17-AAG is dominated by the cytostatic antiproliferative effect that is independent of BAX status.

It was not possible to determine if necrotic cell death was contributing to the overall response of HCT116 *BAX* ^−/−^ tumor xenografts. However, there is increasing evidence suggesting that acute necrosis and the subsequent antitumor immune response generated could have clinical benefit in certain cancers [37, 38]. Unlike cells undergoing apoptosis, necrotic cells lose the integrity of the plasma membrane leading to the release of intracellular substrates that can stimulate fibroblasts and macrophages [37, 38]. These substrates may also act as maturation signals for dendritic cells, which can cross-present antigens to cytotoxic T-cells leading to an antitumor response [39]. An intrinsic limitation of using athymic mice for xenograft studies is the inability to determine the role of the adaptive immune response in the antitumor effect of the drug. Further studies are required to look at this.

During the finalization of the present paper, He et al published complementary data to ours. They also demonstrated that BAX is necessary for the apoptotic response to 17-AAG in vitro but did not characterize the effects of BAX knockout on the activation of alternative cell death mechanisms or the effects of BAX in vivo. The activation of BAX in response to 17-AAG was shown to be via p53-dependant induction of PUMA, which is upstream of BAX [40]. Knockout of PUMA in HCT116 tumor xenografts reduced the apoptotic and overall tumor response to treatment with 17-DMAG, a water-soluble analogue of 17-AAG [40]. PUMA is a proapoptotic member of the BCL2 family that interacts with the antiapoptotic members (BCL-2, BCL-_XL_, MCL1, BCL-_W_) to inhibit their interaction with BAX and BAK [41]. Our own in vivo findings with BAX knockout suggest that the difference in tumor response observed by He et al may not solely be through the loss of PUMA-mediated BAX activation, but potentially through the combined loss of BAX and BAK activation coupled with the loss of repressive activity against the antiapoptotic BCL2 family members.

Our findings have significant clinical relevance, especially because certain cancers, including colon and gastric, are predisposed to loss of BAX expression owing to frameshift mutations in both alleles of the *BAX* gene as a result of failure of DNA mis-match repair systems and microsatellite instability [42]. This genotype is likely selected for to reduce apoptosis during tumorigenesis. Thus it was a concern that HSP90 inhibitors may not be as effective in such patients. Our results suggest that this will not be the case. However, evaluating patient response in clinical trials of HSP90 inhibitors in relation to tumor BAX status and also to the mechanism of antitumor activity (apoptosis vs. necrosis) would be required to truly determine the impact of the observations made in this study on the role of BAX in the clinical response to HSP90 inhibition.

Efforts to increase the anticancer effectiveness and selective tumor cell killing by HSP90 inhibitors include the use of drug combinations [2]. We recently reported the potential for increasing the therapeutic effectiveness of HSP90 inhibitors by decreasing survival signaling and enhancing the apoptotic response in *BAX* ^+/−^ HCT116 colon tumor xenografts by co-administration of TRAIL [43].

In conclusion, we demonstrate that BAX is a critical requirement for the induction of apoptosis in a human colon carcinoma model in response to 17-AAG. In the absence of BAX the overall level of cell death is reduced. Moreover, the lower level of cell death proceeds via a necrotic rather than apoptotic mechanism. Interestingly, overall bulk tumor response to 17-AAG is independent of BAX status, both in vitro and in vivo, which is likely due to the predominance of a cytostatic antiproliferative response combined with the induction of an alternative cell death mechanism when apoptosis is blocked. Our results suggest that BAX status may not alter clinical response to HSP90 inhibitors and indicate that additional drugs may be required in combination to increase tumor-selective cell killing by these clinically promising drugs, such as combination with TRAIL [43] or HSP70 inhibitors [20]. In addition, there are implications from our work for the use of apoptotic endpoints in the assessment of the activity of molecularly targeted agents.

## MATERIALS AND METHODS

### Cell lines and reagents

The isogenic human colon adenocarcinoma cell lines HCT116 *BAX* ^+/−^ and HCT116 *BAX* ^−/−^ were a generous gift from Professor Bert Volgelstein (Howard Hughes Medical Institute, Baltimore, MD) and have been described elsewhere [11]. HCT116 *BAX* ^+/−^ cells are heterozygous for the *BAX* gene, but express BAX protein from the remaining intact allele. HCT116 *BAX* ^−/−^ cells have had the remaining intact *BAX* allele removed by homologous recombination, and hence do not express any BAX protein. Both cell types were cultured in DMEM (Sigma) supplemented with 10% FBS, 1x nonessential amino acids and 5mM L-glutamine (Invitrogen) in a humidified atmosphere of 5% CO_2_, 95% air at 37°C. Cells in mid-log phase of growth were exposed to 17-AAG (Axxora) for 72 hours or the cyclooxygenase inhibitor sulindac sulphide (Calbiochem) for 48 hours. Viability of cells was assessed by trypan blue staining and counting on a hemocytometer. BAX status was confirmed by immunoblotting

### Radiation treatment

Cells were exposed to radiation using a ^60^Co source with a source-to-flask distance of 40cm^3^ and a dose rate of 1.5Gy min^−1^

### Cell growth inhibition

Cells were seeded into 96-well microtiter plates (1.6 × 10^3^ cells/well) and left to attach for 36 hours. A range of concentrations of sulindac sulphide, 17-AAG, CCT18159 [12] or radicicol (Calbiochem) were added to quadruplicate wells for an exposure period of 96 hours. Cell growth inhibition was measured using Sulforhodamine B (Sigma) and 3-[4,5-Dimethylthiazol-2-yl]-2,5-diphenyltetrazolium bromide (MTT) assays as previously described [12, 13].

### Cell cycle analysis

Cell cycle distribution and sub-G1 populations were analyzed by flow cytometry using propidium iodide staining as previously described [8].

### Morphological analysis

Detached cells were harvested 72 hours after treatment with 5×GI_50_ 17-AAG, washed once in PBS (BDH), fixed and embedded [14]. For light microscopy, 1.0 mm sections were cut, dried onto microscope slides, stained with toluidine blue (TAAB Laboratories), and viewed under a Leitz Diaplan microscope. Images were recorded using a Leica DFC320 digital camera.

### Immunoblotting

Cells were collected and lysed in cell lysis buffer as previously reported [15]. Sample preparation was also as described [16]. Equal amounts of protein and SeeBlue®Plus2 molecular weight markers (Invitrogen) were separated on 4-20% Novex® Tris-Glycine gels by electrophoresis and electrotransfered to 0.2μm pore size nitrocellulose membranes (Invitrogen). Membranes were blocked with casein buffer [16] and incubated overnight with primary antibodies against ERBB2 (Santa Cruz Biotechnology), HSP72 (Enzo life sciences), BAX (BD Pharmingen), PARP (Intact and cleaved forms, C-2-10, Clontech), cleaved PARP (Cell Signaling Technology Inc.), BAK and GAPDH (Millipore). Specific antibody-antigen complexes were detected with horseradish peroxidase-conjugated sheep anti-mouse or donkey anti-rabbit IgG (GE Healthcare) and Supersignal West Pico Chemiluminecscent substrate (Thermo Scientific Pierce).

### Tumor xenograft studies

Procedures involving animals were approved by The Institute of Cancer Research's ethics committee and were consistent with published guidelines [17]. Subcutaneous xenografts of the HCT116 *BAX* ^+/−^ and HCT116 *BAX* ^−/−^ human colon adenocarcinoma cell lines were established bilaterally in the flanks of NCr athymic mice. When tumors were of 5 to 6mm in mean diameter, mice were treated with either vehicle (43% ethanol [200 proof], 33% propylene glycol, 24% cremaphor) or 80 mg/kg once daily 17-AAG on days 0-4, 7-11 and 14. Tumor growth was monitored by caliper measurement. For biomarker analysis, tumor samples were taken from a subset of mice on day 5. Protein lysates were prepared as previously described [18].

### Immunohistochemistry and quantitative image analysis

Formalin-fixed paraffin-embedded (FFPE) xenograft tumors were immunostained with Ki67 (MIB-1) monoclonal mouse antibody (Dako) at a 1:2000 dilution and cleaved caspase-3 (Asp175) rabbit polyclonal antibody (Cell Signaling Technologies) at a 1:200 dilution. Standard polymer and avidin–biotin–peroxidase complex techniques were used respectively for immunohistochemistry, which was carried out on 4-μm sections of FFPE tissue. Heat antigen retrieval in citrate buffer at pH 6.0 was applied. Negative controls were included in every run. Slides were counterstained with Harris hematoxylin. Slides stained for Ki67 and cleaved caspase-3 were scanned with an Aperio ScanScope XT (Aperio) using a 20x objective. Whole slide images were analyzed with the Aperio nuclear IHC algorithm (Aperio).

### Statistical analysis

Statistical significance was calculated using Student's two-tailed t-test.

## Supplementary Figures


