# Supplementary material for: Mode of cell death induced by the HSP90 inhibitor 17-AAG (tanespimycin) is dependent on the expression of pro-apoptotic BAX
Source: Oncotarget. 2013 Sep 29;4(11):1963–75. doi: 10.18632/oncotarget.1419 (PMC3875762; doi:10.18632/oncotarget.1419)
Supplement: Supplementary file 1 [file oncotarget-04-1963-s001.pdf]

Mode of cell death induced by the HSP90 inhibitor 17-AAG (tanespimycin) is dependent on the expression of pro-apoptotic BAX – Powers et al

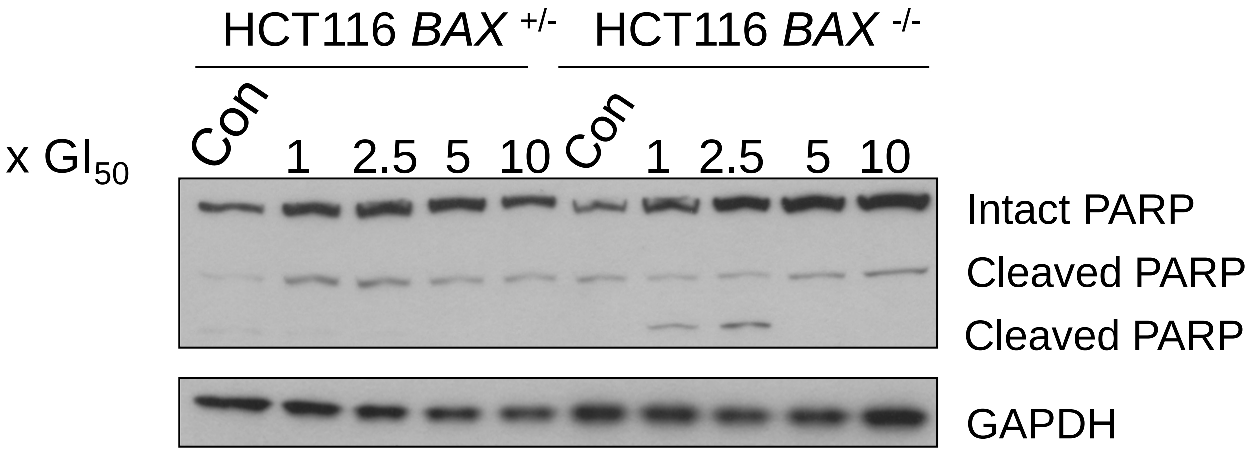

Supplemental data Figure 1: Apoptotic versus necrotic PARP cleavage was detected in attached cells following 17-AAG treatment. Exponentially growing HCT116 cells were exposed to increasing concentrations of 17-AAG at multiples of the individual GI_{50} values for each cell type (HCT116 *BAX* $^{+/-}$ GI_{50} 41.3nM ± 2.3 SEM, HCT116 *BAX* $^{-/-}$ 32.3nM ±1.3 SEM, as determined by 96 hours SRB assay) or the equivalent volume of drug vehicle (DMSO). Cells were harvested 72 hours after the start of treatment and the expression of PARP in cells that remained attached following 17-AAG or DMSO exposure was determined by immunoblotting using an N-terminal specific antibody (C-2-10). GADPH was included as a loading control.
